# Clinical Approaches to Cultural Diversity in Mental Health Care and Specificities of French Transcultural Consultations: A Scoping Review

**DOI:** 10.3389/fpsyt.2020.579147

**Published:** 2020-10-27

**Authors:** Laura Carballeira Carrera, Sarah Lévesque-Daniel, Rahmeth Radjack, Marie Rose Moro, Jonathan Lachal

**Affiliations:** ^1^Alicia Koplowitz Short-Term Fellowship, Madrid, Spain; ^2^APHP, Hôpital Cochin, Maison de Solenn, Paris, France; ^3^Centre integré universitaire de santé et de services sociaux de l'Est-de-l'île-de-Montréal (CIUSSS) du Nord-de-l'Île-de-Montréal, Université de Montréal, Montréal, QC, Canada; ^4^Université Paris-Saclay, UVSQ, Inserm, CESP, Team DevPsy, Villejuif, France; ^5^Université de Paris, PCPP, Boulogne-Billancourt, France

**Keywords:** transcultural psychiatry, migrant families, mental healthcare, psychiatric care, cultural diversity

## Abstract

**Background:** Cultural context substantially affects the experience and clinical expression of psychiatric diseases, while cultural factors limit both access to and effectiveness of care, especially for migrant families requiring specific types of services. We conducted a scoping review on psychiatric services adapted to cultural diversity, to compare these models of care to the French Transcultural Psychotherapy model.

**Methods:** Systematic electronic search of databases (PubMed and PsycINFO), manual search of archives of journals dealing with transcultural psychiatry, and consultations with international experts, to identify all papers describing clinical models devoted to psychiatric care for migrants, published between January 1990 and October 2018. Narrative synthesis of the included articles.

**Results:** The study included 29 papers. The specificities of psychiatric services for migrant families are linked to the host country's migration patterns and citizenship model. In English-speaking countries, specialized services for ethnic minorities offer ethnic matching of the therapist and patient. In Canada, indirect transcultural consultation services have existed since the late 1990s. Australia emphasizes the networking of consultation services and professional training in cultural competence, while the Nordic countries (Sweden, Finland, Norway, and Denmark) focus management on trauma. In France, psychotherapy services, with flexible numbers of therapists involved according to the situation, have existed since 1990.

**Discussion:** Most initiatives place emphasis on training and supervision, in an indirect approach not specifically focused on the patient, or offer cultural matching of patient and therapist. The French transcultural approach, on the contrary, makes the family's culture and its cultural diversity an integral part of the therapy process.

## Background

Julian Tudor Hart set forth his famous Inverse Care Law in the *Lancet* (1971) (1): “The availability of good medical care tends to vary inversely with the need for it in the population served.” That is, the people who need medical care most receive the least, while its availability is concentrated in the population groups that need it least. Migrants and ethnic minorities are the casualties of this systemic inequality in access to the healthcare system, especially to psychiatric facilities. Reports from the World Health Organization (2) and humanitarian groups such as Doctors of the World (3) reveal that the resources dedicated to psychiatry remain inadequate, are distributed unequally, and used inefficiently. These organizations therefore seek to promote the development of public policies to reverse this situation—indisputably needed in this ever more globalized world, where migrants currently account for hundreds of millions of the world's population (4).

At the macro level, this population faces problems including a lack of health insurance coverage, lack of knowledge of the healthcare system, and linguistic barriers. At a micro level, its members run up against the lack of understanding, prejudices, and negative attitudes of many professionals (5). A therapist, for example, may be less interested in and devote less effort to an intervention with a patient perceived as not cooperating or as having a different system of values and with whom the therapist finds it harder to identify culturally. This affects the quality of the intervention, reproducing the Inverse Care Law (6).

This inequality in access to care has consequences at several levels. On the one hand, migrant families and ethnic minorities underutilize the primary healthcare system, at the same time as they overuse emergency departments. On the other hand, these issues can impede the professionals' understanding of the particular psychopathology and can lead to differences in the prescription of drugs, decisions about hospitalization, availability of psychotherapy, and course under treatment (7, 8).

Added to that are pre- and post-migration factors that act as social determinants of mental health: exposure to violence and traumatic migration experiences, the process of acculturation, situations of loss and mourning, adverse socioeconomic conditions, conflicts due to cultural differences, discrimination, and social isolation (3, 8–10).

Finally, it is appropriate to note the major influence of cultural factors on the ways that diseases and their treatments are conceptualized. In every culture, the manner in which symptoms are experienced and interpreted is part of its systems of meaning, and these meanings will model the ways people in those cultures become sick, or cope with feelings of unease, or seek help (11).

There are two positions about how to work with the cultural diversity of families in the organization of health care. The first expects migrant patients to adapt to conventional care, by normalizing or ignoring the differences. The second position recognizes and seeks to remedy these differences by the development of culturally sensitive approaches and clinical practices. Unfortunately, *culture* is often mentioned only when there are misunderstandings or at least difficulties in mutual comprehension between the professional and the patient or lack of adherence to treatment. *Culture* then designates a thing that belongs only to the patient and that represents an obstacle to communication and cooperation (8).

Healthcare systems can adapt in several ways (Table 1): using interpreters and cultural mediators, training professionals in cultural competence and supervision, making innovations in the therapeutic framework of general psychiatric services, and developing specialized clinics for ethnic minorities (8, 10, 12–19). In France, a complete psychotherapeutic method was conceived in the 1980s by T. Nathan and then expanded by MR Moro (one of the authors) for the management of migrant families facing issues that cannot be solved in standard psychotherapy: Transcultural Psychotherapy (TPT). As therapists applying this method, we have reflected at length on the French model, its specificities, and its construction inside the French sociocultural context.

**Table 1:** Definitions.

*Cultural competence*	There are several definitions of this term in mental health care. A review of the literature (1) defines this concept as the skill set that enables professionals to provide culturally appropriate care. This includes consideration of differences due to language and to cultural influences on the expression of distress and ways of seeking help. Other aspects mentioned include respect for the patient's beliefs, as well as a disposition, even a real desire, to learn about other cultures.
*Cultural formulation*	DSM-5 introduced a cultural formulation interview. This evidence-based tool is composed of a series of questions that assist clinicians in making person-centered cultural assessments to inform diagnosis and treatment planning.
*Decentering*	Decentering refers to the ability to distance oneself from oneself and from one's own cultural point of view (2).
*Etiological theories*	The etiological theories refer to the *traditional* explanations for the disease found in non-Western societies—imputed to nonhuman invisible beings (3). Transcultural psychiatry proposes to use these traditional etiologies as a therapeutic lever in their function as cultural containers. In situations of distress or disease, the subject looks to “make sense of the senseless” (4) or of “misfortune” (5). The use of etiological theories belonging to patients allows them to participate actively in the search for meaning and for a solution to the symptoms and their various forms of distress.


We propose to conduct a scoping review to list and describe the multiple ways of providing psychiatric healthcare adapted to cultural diversity and compare these models to the French Transcultural Psychotherapy model, which is the only to propose a complete psychotherapeutic method.

## Methods

This is a scoping review of the international literature on clinical approaches to cultural diversity in psychiatric care.

### Search (Figure 1)

We used three different strategies for the literature search.

**Figure 1:**
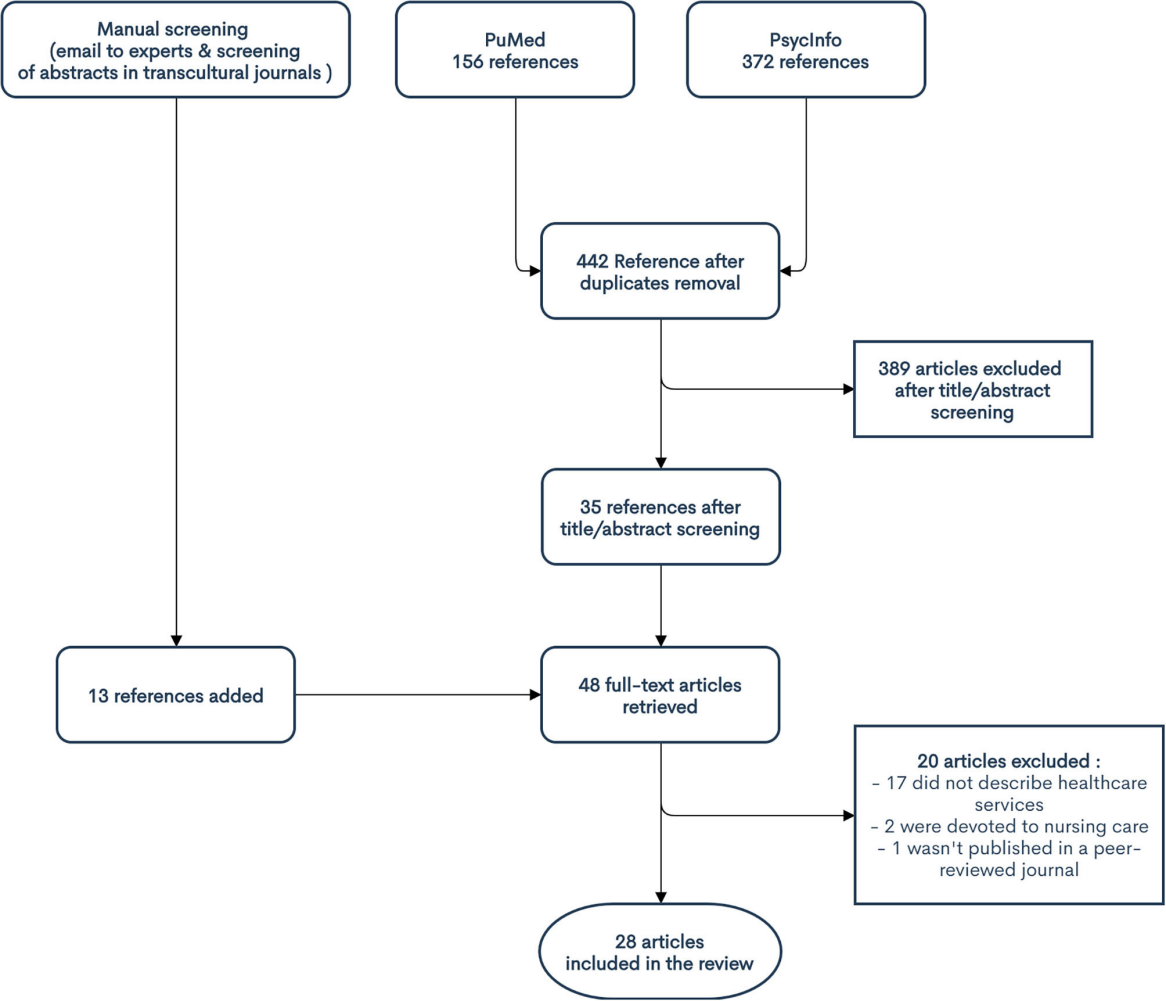
Flowchart of the studies included.

First, on October 1, 2018, we conducted a systematic search for articles identified in the PubMed and PsycINFO databases in response to the Boolean queries in Table 2.

**Table 2:** Boolean queries for database searches.

	(“cultural”[Title] OR “transcultural”[Title] OR “intercultural”[Title] OR “cross cultural” [Title]) (PubMed) (“cultural”[TI] OR “transcultural”[TI] OR “intercultural”[TI] OR “cross cultural” [TI]) (PsychInfo)
AND	(“migrants” OR “ethnic minorities” OR “cultural diversity”)
AND	(“psychiatrist” OR ”mental health”)
AND	(“care” OR “services” OR ”treatment”)

After duplicates were removed, 592 titles and abstracts were screened by two researchers (LCC & JL). The inclusion/non-inclusion criteria were the following:

Peer reviewed journal articles (to ensure enough quality on the selected papers

Dealing specifically with clinical models for handling cultural diversity in psychiatric treatment (articles which only referred to transcultural psychiatry at a theoretical level were discarded)

Referring to psychiatric healthcare services that provide care adapted to cultural diversity

Describing the modalities of care

Published between January 1, 1990, and October 1, 2018

In English, French, or Spanish.

Thirty-five full texts were retrieved.

Second, we asked within our international network of transcultural therapists (and researchers) for references to provide any journal article that met these inclusion criteria.

Finally, the research was completed by an analysis of the abstracts of the two principal journals in the national and international transcultural field: *L'autre* (French) and *Transcultural Psychiatry* (English).

These methods identified an additional 13 full-text papers.

After full-text screening, 29 papers were finally included in the review.

### Quality Appraisal

All the papers included were conceptual articles, and we found no existing checklists by which we could systematically assess their quality. To improve the rigor of our review, we created a simple tool to assess the quality of each paper, shown in Table 3. After the research group constructed the appraisal tool, two researchers (LCC and JL) independently evaluated each paper and the working group reached a consensus about it. Fleiss Kappa was used to measure inter-rater agreement using R software 4.0.2.

**Table 3:** Evaluation of the quality of the studies—graded from 0 (absent) to 5 (clear).

**References**	**The authors' affiliations are clear**	**The international context of migration and mental health policies is adequately described**	**The national context of migration and mental health policies are adequately described**	**The health services available are identified**	**The description of the available health services is adequately clear**	**The authors discuss the limitations of the health services available**
(6)	5	2	5	5	4	4
(7)	5	4	5	5	4	5
(8)	5	4	5	5	5	3
(9)	5	0	2	5	4	3
(10)	5	0	5	5	5	5
(11)	5	5	1	5	5	5
(12)	5	3	4	5	5	4
(13)	5	0	2	4	4	2
(14)	5	2	5	3	2	4
(15)	5	2	5	5	5	5
(16)	5	0	4	5	5	5
(17)	5	0	3	3	2	5
(18)	5	0	4	5	5	5
(19)	5	2	3	5	5	5
(20)	5	2	3	5	5	5
(21)	5	0	5	5	5	5
(22)	5	0	5	5	5	5
(23)	5	0	5	5	5	5
(24)	5	5	4	5	5	5
(25)	5	3	5	5	5	5
(26)	5	0	1	3	2	1
(27)	5	0	5	5	5	5
(28)	5	4	5	5	5	5
(29)	5	0	4	5	5	3
(30)	5	0	2	5	5	4
(31)	5	3	4	4	3	4
(32)	5	3	5	5	5	5
(33)	5	3	5	5	5	5
(34)	5	0	5	5	5	5
*kappa*	*1*	*0.813*	*0.789*	*0.75*	*0.638*	*0.818*

### Analysis

The articles were read by three authors (LCC, JL, and MRM), and summarized by the first author. The results and their presentation were then discussed during research group meetings. To synthesize them, we looked for similarities between the care models described in the different papers and organized the results inductively. They nonetheless reflect, as previously described in the literature (20), the association of countries' histories, patterns of migration, citizenship models, and particularities in the development of psychiatric services for immigrant populations. The French model is presented in a second part of the results and is then discussed in relation to the different models in the discussion.

## Results

This review includes 29 articles. Table 4 presents the principal characteristics of the articles, and Table 5 the principal characteristics of the services and care that the articles describe. Table 6 details the categories of “adaptations of the healthcare system” used by the authors, according to their similarities.

**Table 4:** Characteristics of the studied included.

**References**	**Year**	**Country**	**Aim of the study**	**Type of adaptation of the healthcare system**	**Main results**	**Main conclusions**
(6)	2016	UK	It aims to explore the role of the “transcultural mental health worker” as an alternative to the use of interpreters	Making innovations in the therapeutic framework of general mental health services: Introducing a ‘Transcultural Mental Health Worker'	Participants found working with transcultural mental health workers either more effective than or as effective as interpreters.	The study highlights the importance of providing linguistically and culturally appropriate treatment modalities for minority ethnic patients
(7)	2012	Sweden & Germany	To provide an overview and a critical examination of current conceptualization of cross-cultural mental health training.	Cross-cultural competence training programs and supervision	Description of the adaptations made in Sweden and Germany	The authors criticize “cultural competence” as it is conceptualized and propose different ways for “cross-cultural” training
(8)	2013	Sweden, Norway & Germany	To examine cultural diversity in the countries in question, discuss challenges and give examples of current work to open up mental health services to cultural diversity	(i) Cultural competence training for professionals and (ii) Developing specialized services for ethnic minorities	In all three countries, some efforts have been made to provide culturally sensitive mental health services	The development of culturally-sensitive care requires institutionalized support, long-term funding, research, guidelines and training of staff
(9)	2014	Australia	To explore the experience of clinicians who are vicariously exposed to trauma, because they support people from refugee- and asylum-seeking backgrounds	Developing specialized clinics: Services to refugees, asylum seekers, displaced persons, or migrants who have experienced torture and trauma	The findings demonstrated that clinicians can be positively and negatively affected by their work and that mobilizing appropriate coping strategies can help to minimize distress	A number of challenges can affect clinician well-being supporting refugees, and care must be taken to ensure that the risks of trauma work are minimized
(10)	2015	UK	This paper reports on a feasibility study and evaluation of a new type of cultural consultation service (CCS)	Cultural competence training for professionals	The CCS model of care improved service-user experience and recovery, workforce development, and saved costs on out- patient contacts, while improving overall functioning in the most complex cases	Overall, the CCS seemed acceptable and the implementation and use of the service by patients showed feasibility for future work
(11)	1993	New Zealand	This article describes how cultural differences are addressed by the Regional Forensic Psychiatric Services of Auckland and Northland	Making innovations in the therapeutic framework of general mental health services: Community and Liaison Services, with access to 'cultural advisers' from the indigenous community	They describe how cultural differences are addressed by departments, through liaison with the local tribes and the active recruitment of culturally representative staff	Efforts are being made in New Zealand to address cultural differences in institutions. To this end, alternative cultural services are being established by tribal groups. When this separate provision of services is not possible, implementation of biculturalism is being made to provide high-quality services to patients.
(12)	2005	Netherlands	The purpose of this article is to present a model to promote and assess interculturalization of mental healthcare services	(i) Training professionals in “ethnocultural sensitivity” and (ii) Making innovations in the therapeutic framework of general mental health services: Interpreters, culture-brokers and cultural consultants services	The model describes qualitative and quantitative criteria and indicators to be applied in the different contexts	They are uncomplicated tools to evaluate the inter-culturalization process of mental health services
(13)	2014	Australia	This paper considers the processes by which evidence-based interventions can be adapted by health professionals in any context; and includes an example of a successful cultural adaptation to an evidence-based intervention	Cultural competence training for professionals	They describe the Aboriginal Mental Health First Aid course program, a training for professionals to assist people with mental health issues from culturally diverse backgrounds	The paper concludes by outlining the steps mental health professionals can take when adapting evidence-based interventions for use in their own work- place settings
(14)	2013	Nordic Countries	This article discusses major themes in recent transcultural psychiatric research in the Nordic countries from: (a) epidemiological studies of migration, (b) indigenous populations, and (c) quality of psychiatric care for migrants	Developing specialized clinics for trauma-affected refugees and ethnic minorities	Varied communication patterns and cultural and linguistic differences complicate the process of diagnosing and treating patients from immigrant backgrounds. Qualitative research methods may be most appropriate for exploratory studies in this emerging field.	This review underlines the need for formalized education and training of clinical staff
(15)	2005	UK	It describes several projects that have been developed within the NHS in different parts of England	Developing specialized services for ethnic minorities	There are a variety local projects incorporating good practices, but there is no overall consensus on a single good practice model	Sustainable changes require a national plan and strategy to promote innovations in general mental health services
(16)	2005	Canada	This article is a description of how cross-cultural services in mental health have evolved in Vancouver	(i) Developing specialized services for ethnic minorities; (ii) Cultural competence training for professionals; and (iii) Making innovations in the therapeutic framework of general mental health services: Interpreter Services and Multicultural Liaison Program	The cross-cultural mental health service has experienced increased coordination under the regional health services administration	Vancouver cross-cultural mental health system provides health care that is accessible, available and acceptable to all, and develops health care that acknowledges racial and cultural diversities
(17)	2017	Netherlands	This article presents a study on the feasibility, acceptability, and utility of the Brief Cultural Interview (BCI), with refugee and asylum-seeking patients in a Dutch center for transcultural psychiatry	Developing specialized services for ethnic minorities, refugees, and asylum seekers	This study has given evidence that, without losing its usefulness for the sake of valid diagnosis, the BCI (Brief Cultural Interview) is more feasible than the longer CFI (Cultural Formulation Interview)	The promising results of these pilot data could offer an encouraging impulse for the culturally sensitive treatment of mental health problems, though it requires confirmation from other studies around the globe
(18)	2012	USA	This article presents a pilot program, the “Psycho-Social-Cultural Treatment Group” for Cambodian refugees	Making innovations in the therapeutic framework of general mental health services: Psycho-Social-Cultural Treatment Group	This program was developed by combining Cambodian cultural traditions and spiritual philosophy with Western mental health techniques	It is suggested that the current pilot programs might contribute to helping professions in community-based agencies that work with traumatized Cambodians
(19)	2003	Canada	This paper reports results from the evaluation of a cultural consultation service (CCS) for mental health clinicians	Making innovations in the therapeutic framework of general mental health services: Various forms of cultural consultation	Cases seen by the CCS clearly demonstrated the impact of cultural misunderstandings. Clinicians referring patients to the service reported high rates of satisfaction with the consultations	The cultural consultation model effectively supplements existing services to improve diagnostic assessment and treatment for a culturally diverse urban population. Clinicians need training in working with interpreters and culture brokers.
(20)	2013	Australia	This paper describes the development of a pilot secondary consultation program by a state-wide transcultural psychiatry unit	(i) Cultural competence training for professionals and (ii) Making innovations in the therapeutic framework of general mental health services: Cultural consultation services	Participants from a range of disciplines provided consistently positive feedback. The sessions provided an effective forum for discussing cultural formulations and culturally sensitive approaches	This pilot study facilitated the development of cultural consultation services
(21)	2005	Canada	To look at the specificities of the work of a Transcultural Child Psychiatry Team developed in Montreal	Making innovations in the therapeutic framework of general mental health services: Transcultural Psychiatry Team	The authors explain the clinic's development and method of service provision for its patient population. They also describe the Transcultural Child Psychiatry Team and its modalities of assessment and treatment	In this model of service delivery, current mental health care practice is modified in order to address the social specificities and cultural diversity of transcultural child psychiatric populations
(22)	2009	Canada	This article describes a preliminary qualitative evaluation of a mental health program in a multi-ethnic environment in Montréal	Making innovations in the therapeutic framework of general mental health services	A preliminary qualitative evaluation of the project showed the numerous sources of uncertainty which participants face	The authors formulate the hypothesis that uncertainties, although they generate uneasiness and confusion, also enable an opening toward innovation and otherness
(23)	2006	Canada	This article examines how the issue of clinical intervention with the Aboriginals presents itself within Montreal's transcultural psychiatric services	Making innovations in the therapeutic framework of general mental health services: Transcultural Psychiatry Team	The author questions the place and response given to the demands of a minority unlike the others, the Aboriginals	The creation of transcultural clinics is a recognition by public health departments of the importance of the specificity of the social contexts, value systems, and interpretations of reality of diverse cultural groups
(24)	2013	UK	This article presents the preliminary results of the implementation of a cultural consultation service (CCS)	Cultural competence training for professionals	Results indicate that clinicians developed a broader and patient-centered understanding of culture, and gained skills in narrative-based assessment method, management of complexity of care, and clinical cultural formulation	They conclude that cultural consultation model is an innovative way of training clinicians in cultural competence skills
(25)	1990	Australia	It describes the development of a Service for the treatment and rehabilitation of torture and trauma survivors (STARTTS)	Developing specialized services	The experience of staff and responses of survivors to therapy have illuminated a number of issues that are central to the delivery of an effective and accessible service of this kind	Recognition of the importance of professional training and clinical expertise in working with disturbed survivors of torture and trauma
(26)	2007	Spain	The aim of this preliminary research was to create several forms appropriate to a specific model of medical attention for the mental health for people culturally different and/or at risk of social marginalization	(i) Cultural competence training for professionals and (ii) Making innovations in the therapeutic framework of general mental health services: Supervision and consultation services	This model defends and tries to put into practice a sort of assistance in which special attention is paid to every cultural, social, political or economic variable that can be related to people's mental health	The representations of health and disease determine in a very significant way both the Medical Care Process and the population's levels of health
(27)	2017	Australia	This article describes the expansion of a transcultural secondary consultation model run by a state-wide transcultural unit	Making innovations in the therapeutic framework of general mental health services: Cultural consultation services	The results emphasize the need for multidisciplinary collaboration and a facilitated space for clinical teams to explore culturally responsive therapeutic practices	The discussion highlights the usefulness of a transcultural model of consultation and identifies the benefits the model brings to understanding and intervening with clients, culture, and systems
(28)	2017	Belgium	This article proposes an intervention methodology that improves access to and the effectiveness of mental health care facilities for children and their families with backgrounds as refugees and migrants	Support of a non-profit organization, specialized in refugee care, embedded in a psychiatric unit	This intervention has a positive influence on creating healing ties between parents and care providers	There is a need for further research on the effectiveness of transcultural interventions, which would allow for a more structural implementation of them
(29)	2011	USA	The aim of this study was to identify components of cultural competence in mental health programs developed for cultural groups by community and mental health professionals from these groups	(i) Cultural competence training for professionals and (ii) Making innovations in the therapeutic framework of general mental health services: Family and community involvement	Components included communication competencies; staff in culturally acceptable roles; culturally framed trust building, stigma reduction, friendly milieus and services; and peer, family, and community involvement	Incorporating these components into any program in which underserved cultural populations are seen is recommended for improving cultural competence
(30)	2015	Australia	This paper describes the STARTTerS Early Childhood program (Service for the Treatment and Rehabilitation of Torture and Trauma Survivors)	Making innovations in the therapeutic framework of general mental health services: A model for collaborative and consultative design and implementation of culturally appropriate services	This research has led to ongoing collaborative and consultative processes, resulting in the development of services and referral systems, which will build a comprehensive and culturally appropriate early childhood program	Systemic programs as community consultation, research, cross-referral and liaison with other service providers can be used to provide appropriate mental health care
(31)	2006	Canada	It describes the development of the transcultural project of Jean-Talon Hospital (Montréal)	Making innovations in the therapeutic framework of general mental health services	It provides an historic perspective of the transcultural approach and the context of Jean-Talon project development	The author gives some ideas on how Québec could contribute to the future development of ethnopsychiatry
(32)	2011	Italy	It aims to evaluate the effectiveness of “Bologna West Transcultural Psychiatric Team” and to describe the characteristics of patient and psychiatric interventions related to dropping out.	Making innovations in the therapeutic framework of general mental health services: Consultation liaison activities to improve cultural competence	The strongest predictors of dropping out were non-Asian origin, a recent history of migration, and not receiving social intervention.	Psychiatric consultation services to migrants could be made more effective by enhancing: (a) cultural competence, through cultural mediator involvement; and (b) social support from the first psychiatric contact
(33)	2009	Italy	The aim of this study was to investigate the socio-demographic and clinical features of immigrants referred to the Bologna-West Transcultural Psychiatric Team	Making innovations in the therapeutic framework of general mental health services: Consultation liaison activities to improve cultural competence	Clinical diagnoses, psychopathology and pathways to care were closely related to socio-demographic features and ethnic group	More efforts should be made to ameliorate pathways to care among migrants
(34)	2005	USA	Qualitative assessment of cross-cultural mental health services in California	(i) Ethnic matching and (ii) Cultural competence training for professionals	Ethnic matching (whereby the ethnicity of the patient is matched with the ethnicity of the clinician) and cultural-competency training emerged as prevalent strategies to address patient diversity.	Strategies commonly used to improve culturally appropriate mental health care are often inadequate to meet the needs of diverse communities. New strategies such as a Cultural Consultation Service are needed to supplement existing services

**Table 5:** Description of the international transcultural services.

	**Country**	**Types of services**	**Description**	**Cited in**
Substantial immigration from former colonies	UK	Services for minority ethnic communities	Transcultural Psychiatry Unit at Bradford, the Maudsley Outreach Support and Treatment Team, the North Birmingham Home Treatment Service, the volunteer organization “*Ipamo*”	(15)
		Cultural Consultation Service	The CCS is an innovative model to promote cultural competence of clinicians and directly improve patient experiences and outcomes from care	(10, 35)
		Transcultural Mental Health worker	Use of transcultural mental health workers as an alternative to interpreters in an attempt to identify the communication barriers and improve the mental health care for black & minority ethnic patients	(6)
Multicultural citizenship model countries	USA	Mental Healthcare for minority ethnic communities	Mental Health clinics for ethnic minorities or ethnic matching between clinician and patient	(34)
		Psycho-Social-Cultural Treatment Group	Specialized program for refugees, combining their cultural traditions and Buddhist philosophy with Western mental health techniques	(18)
		Cultural Competence	National Center for Cultural Competence: to design, implement, and evaluate culturally and linguistically competent service delivery systems to address growing diversity, persistent disparities, and to promote health and mental health equity	(29, 36)
	Canada	Transcultural Psychiatric Teams	Transcultural Psychiatric Teams of Jean-Talon Hospital; Montreal Children Hospital	(21, 23, 31)
		Projet de soins concertés en santé mentale jeunesse dans un milieu pluriethnique (joint care project for youth mental health in a multiethnic environment)	Partnership between the transcultural psychiatry team at Montreal Children's Hospital and professionals at local community centers (CLSC) and youth centers	(22)
		Cross Cultural Mental Health Services	A culturally responsible approach to diagnosis and treatment. Clients' cultural and language needs are matched with a staff member who can speak the language and/or is familiar with the culture.	(16)
		Cultural Consultation	Various forms of cultural consultation, including direct assessment, secondary consultation and discussions with community organizations about cross-cultural aspects of mental health	(19)
	Australia	Transcultural Consultation Program	It assists mental health services through workforce training and service development with the aim of improving the quality of care provided to individuals and families from culturally and linguistic diverse backgrounds	(20, 27)
		Aboriginal Mental Health First Aid course program	It is a structured program for professionals and non-professionals who seek to be better prepared to assist people with mental health issues from culturally diverse backgrounds	(13)
		Non-for-profit organization for refugees and migrants	It provides psychological services to refugees, asylum seekers, displaced persons, or migrants, who experienced torture and trauma before migrating to Australia	(9)
		Service for the Treatment and Rehabilitation of Torture and Trauma Survivors	STARTTS: Service for the treatment and rehabilitation of torture and trauma survivors STARTTerS: Early Childhood Programme at the NSW Service for the Treatment and Rehabilitation of Torture and Trauma Survivors	(25, 30)
	New Zealand	Community and Liaison Services	Community and Liaison Services, with access to 'cultural advisers' from the indigenous community	(11)
Other European countries with diverse initiatives	Sweden	Transcultural Center	The Transcultural Center in Stockholm supports health professionals by training, supervision and consultations, networking, knowledge transference and support of local clinical developmental work	(8, 36)
	Norway	National Center for Mental Health for the indigenous population	The Sámi National Center for Mental Health	(36)
	Denmark	Competence Center for Transcultural Psychiatry	A specialist outpatient clinic forming part of the Mental Health Services for trauma-affected refugees. Aims: to provide treatment, and research on transcultural psychiatry	(14)
	Italy	Transcultural Psychiatric Team	Specifically designed to ameliorate cultural competence through consultation liaison activities that encompass not only primary care facilities, but also social services and voluntary organizations	(32, 33)
	Germany	Cross-cultural *opening* of the health care system	Cross-cultural competence training Multicultural staff recruitment Implementing culturally and linguistically specialized treatment programs	(36)
	Spain	Transcultural Psychiatry Program	Specialized team on a mainstream healthcare service, offering consultation, supervision, and cultural competence training for professionals; mental health care for migrants	(37)
	Netherlands	*Interculturalization* of mental health servicesCenter for Transcultural Psychiatry	De Evenaar is a Center for Transcultural Psychiatry that provides mental health care in the northern part of the Netherlands to migrants, refugees, and asylum seekers. The Center offers several adult and youth therapy programs, both inpatient or outpatient care	(12, 38)
	Belgium	Psychiatry Assisting a Cultural diverse Community in creating healing Ties (PACCT)	Non-profit organization that provides mental health care for children and their families with a refugee and migration background	(28)

**Table 6:** Type of adaptation of the healthcare system.

• Making innovations in the therapeutic framework of general mental health services
Principal initiatives:
- Transcultural Psychiatry Consultations
- Interpreters and culture-brokers services
- Ethnic matching
- Cultural Consultation Services
Others: Community and Liaison Services, with access to 'cultural advisers', introducing a “Transcultural Mental Health Worker,” family and community involvement.
• Developing specialized services
- For ethnic minorities
- For refugees, asylum seekers, displaced persons, or migrants who have experienced torture and trauma
• Adaptations aimed for professionals:
- Cultural competence training and supervision for professionals

The results of the critical appraisal are reported in Table 3. The quality of the articles was globally good, except for the criterion concerning the description of the international context of immigration policies. No article was excluded solely on the basis of inadequate quality.

The adaptation of psychiatric care to a context of cultural diversity began in English-speaking countries in the 1970s and in France in the 1980s (12, 14).

### Different Types of Services Developed Across the World

In this first part, we sketch the different international models described in the literature. As stated above, these models have been regrouped according to their similarities and they reflect the countries' histories, patterns of migration, and citizenship models (20).

First, we find countries such as the United Kingdom and France, which have had substantial immigration from their former colonies. These migrant populations very often faced racism and discrimination on their arrival.

In England, transcultural psychiatry began to develop at the end of the 1970s, with the creation of specialized services for ethnic minorities. Later, professionals were introduced to concepts such as cultural sensitivity, antiracist practices, and *misdiagnosis* (diagnostic errors due to the failure to take cultural factors into account). Multicultural and multidisciplinary advisory teams appeared, and professionals of varied cultural origins were recruited (12). More recently, the United Kingdom has developed an innovative model: the Cultural Consultation Service (CCS). This is an adaptation of the model developed at McGill University in Canada (and described more fully below), which uses an ethnographic methodology and is based on medical-anthropological knowledge. These departments aim to improve the evaluation, treatment, and outcome of immigrant families. They also seek to act on the structural determinants of inequality in access to psychiatric care and increase the cultural competence of professionals (17, 18). Various practices are therefore recommended without any general consensus around a single model (12).

Some countries, such as the United States, Canada, and Australia, whose populations were shaped by successive waves of migrants, have a multicultural citizenship model. This model promotes the existence of multiple cultural communities within the society. These countries thus tend to recognize cultural diversity and its stakes for health in general. There are also ethnospecific clinics (20).

The United States is a country that was built largely through immigration, but has also been deeply marked by its history of slavery and racism. Despite the existence of policies promoting assimilation, migration flows have led to the preservation of different cultural communities. The development of ethnospecific clinics is a response to this diversity. In these clinics, the professionals know the language and the culture of the community they serve (20, 21). Ethnic matching of therapists and patients is also facilitated in general medical care (8, 22). Moreover, it is recommended that components of cultural competence be incorporated into any psychiatric program covering cultural minorities (22, 23).

The United States is also where ethnopsychiatry and ethnopsychoanalysis were born, after World War II, at the Menninger Clinic (in Kansas until 2002, when it moved to Texas), which used anthropology and clinical practice complementarily and strongly influenced the principal French model (24).

In Canada, cultural identity is considered fairly positively, and the concept of “reasonable accommodation” is relatively widespread. The law encourages pluralism and diversity to preserve the language and culture of ethnic minority groups and to combat racism (20). Cultural psychiatry has attempted to meet the challenges presented by the diversity of the population in general healthcare facilities, beyond the development of ethnospecific services in some cities. At the beginning of the 1990s, combining the Canadian concepts of “*multiculturalisme de convivence*” (multiculturalism of living together, as opposed to that of dominance) with French ethnopsychoanalytic traditions, several plans for transcultural teams took form in the Montréal region (8, 25, 26). Clinical interventions with multi-ethnic populations and the indigenous are included, as well as a specific Transcultural Child Psychiatry Team (27–29), In 1999, to cope with the limitations of this system, the Cultural Consultation Service (CCS) of McGill University was created. It used a consultation-liaison model, which integrates the medical-anthropological approach and Western psychiatric care. Families are referred by a professional who considers that cultural factors are compromising the evaluation, treatment, or therapeutic relationship. The CCS, with the aid of interpreters and cultural mediators, assembles the information necessary to understand the patients' narratives. The team then researches and drafts a cultural formulation (Table 1), which is submitted to the referring professional, accompanied by treatment guidelines and possible management strategies (8, 30).

In Australia, various services have been developed to meet the needs of ethnic minorities and Indigenous communities. The choice for the Indigenous communities was to give them the control in the development and management of care services. Efforts for migrant families have primarily concentrated on language barriers and cultural competence training for professionals (20, 31). Accordingly, all states and territories in Australia have transcultural mental health resources, funded by the public healthcare system. They make up the Australian Transcultural Mental Health Network, whose function is to support mental health care nationwide, through research, professional training, and innovation in services. Its objective is to improve the accessibility, quality, and cultural appropriateness of mental health care for migrants. Specific innovations include the creation of jobs such as consultant in ethnic mental health and the recruitment of bilingual staff (16, 19, 32). Moreover, specialized services have been developed for the treatment of victims of torture and trauma to help refugees (33, 34).

In New Zealand, cultural differences are addressed through Community and Liaison Services, with access to 'cultural advisers' from the indigenous community (35).

Countries that have not traditionally received large populations of migrants are also now attempting to respond to cultural diversity to provide greater social justice and appropriate care for all patients. In particular, the Nordic countries, which have been culturally homogeneous until recently (except for several indigenous minorities) have experienced an increase in the diversity of their populations. In Sweden, Finland, Norway, and Denmark, special focus has been placed on developing services to treat the sequelae of violence and trauma as well as on training in cultural competence in general healthcare facilities. Psychiatric services specific for indigenous populations have also been set up (5, 36–38).

In other European countries, isolated initiatives have been launched to respond to the increase in cultural diversity. Nonetheless, no government policies have sought to improve the access of migrant families to psychiatric care. Italy, Germany, and Spain have set up teams aimed at providing transcultural training for psychiatric professionals (7, 15, 39, 40). Italy has several transcultural care teams in departments of psychiatry and child psychiatry; not only do they offer consultation-liaison services, but they can conduct psychosocial and psychotherapeutic interventions in the most complex cases (7, 15). In particular, Italy has developed cultural mediators, as in Milan (Crinali) (41). Germany and the Netherlands are trying to guarantee greater cultural openness in public psychiatric facilities (5, 42), while in Belgium this initiative depends more on non-profit organizations (9).

### The French Model: Trans cultural Psychotherapy Services, With Flexible Numbers of Therapists Involved According to the Situation

The French citizenship model tends to minimize the importance of cultural differences in individuals in favor of adherence to the shared values of the Republic. Traditionally, the multiculturalism established in France is one that might be called a multiculturalism “of dominance,” in which cultural identity can be expressed in the private sphere but is not recognized or valued in the public sphere. There is a widely shared fear of migrant communities. For the sake of integration, homogenization of these differences is expected in the public space (25, 26). Therefore, health care in France is traditionally considered to be addressed to everyone, with no specificity linked to their cultural origins and without any recognition of the obstacles that might prevent patients from access to these services, which are theoretically available to all.

Nonetheless, French psychiatrists and psychologists who see migrant patients must deal with the limitations of this concept of care. In the 1980s, the first foundations of transcultural psychiatry were laid in France, based on the ethnopsychoanalytic theories developed by Devereux (43). According to Devereux, the basic mechanisms of mental functioning are universal, but the processes of an individual's socialization in their culture of origin must be understood to be able to access this universal dimension, since these cultural processes generate diverse and varied clinical events (10, 14). From this paradigm, Tobie Nathan at the Avicenne Public Hospital created an innovative psychotherapeutic framework intended for migrant families: the ethnopsychiatry group. Marie Rose Moro, who became director of the program in 1989, modified some elements to adapt it to the children of migrants (the second generation). She insists on the importance of the process of cultural *métissage* (hybridization) and of decentering (Table 1) (14). A group of transcultural therapists is a central element of this flexible service offered to families, and its most original aspect. We will therefore analyze it now, noting that it does not summarize the model, which can also work in small groups or on an individual basis (with or without an interpreter).

This group-based model of transcultural service shares the factors common to all psychotherapy, such as the construction of a narrative, the establishment of a therapeutic relationship, and a variety of specific theoretical and methodological factors (44).

#### Organization of Transcultural Therapy

Transcultural psychotherapy applies a therapy technique based on two complementary interpretations of symptoms rather than a simultaneous reading. Accordingly, anthropological and clinical psychoanalytic approaches are used. The clinical approaches rest on elements from psychoanalytic parent-children therapy, narrative therapy, and systemic and psychoanalytic family therapy, combined with techniques of cultural mediation (14).

Most often, referrals for transcultural management arise during the treatment of children, when medical, social, educational, or other institutions consider that second-line treatment is needed after the failure of standard management. The indication is stated in terms of the complexity of the situation and of the clinical problem, when the team referring the patient considers a cultural clinical approach necessary. In some cases, these referring teams can be seen in an indirect consultation, that is, without the family, to analyze the interventions and help adapt the care strategies (10, 44).

The first consultations are intended to construct the alliance and the treatment plan with the family. Once the plan is constructed, the usual follow-up is then organized in sufficiently long sessions (around 90 min), scheduled every 6–8 weeks.

Patients are invited to bring their families to these consultations. They are received by a group composed of several therapists of diverse cultural origins and an interpreter-cultural mediator of the same culture as the family, who can interpret successively in both directions (patient-therapist or therapist-patient). At least one professional from the referring team, who is managing and knows the patient, is also invited.

The group is multicultural and multilingual. It is directed by a principal therapist and relies on the trained co-therapists. For the management of children, one of the co-therapists becomes the auxiliary co-therapist for the child, by sitting down to play with him or her, in an area set up for this in the center of the group, with a table, crayons, and games to play. The group represents and embodies otherness and makes it possible to transform this otherness into a therapeutic lever. It thus serves as a support for psychological construction (45). The framework of the group functions as a transitional space in the sense used by Winnicott: a space for listening and receiving, enabling patients to talk about their cultural representations, protected from criticism and lack of understanding. The group holds the family and the child—in Winnicott's sense of “holding” (46). It becomes a transitional space: in the face of the cleavage of migration, the group is a mediator that makes it possible to integrate the culture of origin and that of the host country (14). Finally, management by a group is congruent with the collective approach to care found in traditional societies (8, 14, 45, 47).

The transcultural consultation is a flexible system, and the size of the group can be adapted to the situation. The classic large group includes around 10 co-therapists, as well as trainees. Over the years, the transcultural framework has progressively dealt with new domains, including questions of intergenerational transmission, family dynamics, and child development in the context of migration and even adoption (14, 48). The referrals of unaccompanied minors or patients needing specific work around psychological trauma have required some modifications in the size of the group or the function of the co-therapists (10). Experiments with smaller groups have also been proposed according to the family's cultural origin, when large groups have no particular anthropological interest (in families from Southeast Asia, for example), contrary to the families from North Africa and West Africa, who accounted for most families at the time the group system was created and for whom the group has a protective valence that facilitates expression.

The presence of the interpreter is a key parameter in transcultural work, both at the linguistic level (understanding one another) and the symbolic level (recognizing the identity and singularity of the other). The interpreter enables each family member to speak their own native tongue and to recognize its value to themselves and their children, an element that facilitates the construction of their identity (44, 49). It has been shown that this interpreter has a function as much for second-generation children, speaking French, as for the first generation (49).

#### Therapeutic Processes

The objective of transcultural therapy is to promote a creative dialogue and a co-construction of personal and family narratives that lean on the representations and experiences of the patients, whether they are individuals, families, or collective groups. The principal therapist gives the floor to participants and is always the person addressed. This mode of communication, which anthropologists call indirect, enables great emotional containment. During the sessions, the co-therapists speak at the principal therapist's request to propose their hypotheses, representations, or images, relying on their own attachments, history, and culture. They may evoke myths, history, traditions, etc. These references to personal experience open the door to a dialogue about cultural complexity and the different readings possible in situations of cultural *métissage* (hybridization) (10, 14, 44).

On this basis, the group enables the formulation of different conceptions of reality and of what the patient and the family are experiencing. It makes it possible to open the discussion to various—and sometimes divergent—daily realities. This self-disclosure by the group authorizes and supports the family members' self-narrativity. The group accompanies them in a reflexive process in which they can question themselves and transform their subjective representations. Each can thus attain a more flexible and complex self-identification and use all of their skills to find new ways of resolving their conflicts (10, 14, 44, 48).

Finally, the framework enables the emergence of narratives that are difficult to share in the framework of individual therapy. These narratives deal, for example, with migration experiences, questions about cultural *métissage*, and transmission, but also etiological theories about the origin of both the disease and the distress (10) (Table 1). The etiological theories can thus serve as cultural containers that make it possible to ascribe a meaning to the symptoms and to the psychological distress.

The transcultural group opposes an ethnocentric perspective and promotes transcultural encounters. The viewpoint proposed is that of the wealth and multiplicity that results from situations of *métissage*. It thus becomes a space where the dominant cultural discourse can be questioned, with the suspension of the psychiatric diagnosis performed from western classifications (14, 48).

The process of decentering is essential to allow this encounter. One of its techniques involves the analysis of cultural countertransference, defined as therapists' explicit and implicit emotional reactions to the otherness of a patient who belongs to a different culture. Therapists try to be aware of these reactions, most often during work with the group, both before and after the consultation (10, 14, 45, 48). This can also take place later, as part of group seminars where they try to describe and then analyze this cultural countertransference.

## Discussion

Varied models lead to different methods of taking cultural diversity into account in psychiatric treatment. Nonetheless, most of these initiatives have stressed training and supervision—the approach to care is thus indirect and does not take the patient as its object—or the cultural matching of patient and therapist. The aim of these methods is to modify the framework of care, that is, the services provided, to search for a compromise between the patient and the therapist, etc. The French transcultural pychotherapy approach, on the contrary, is a complete psychotherapeutic method aimed at patients rather than at the framework of care.

The models that offer matching between the therapist and patient by language or ethnicity or both present the problem that in fact language, ethnicity, and culture are not equivalent concepts. Even more, the patients have all taken part in an acculturation process that requires the métissage (mixing or blending) of ways to think about distress. In the French transcultural pychotherapy approach, the therapists of the group are not experts in the patient's culture, but rather in the very concept of culture and cultural attachments. The therapeutic work, which involves understanding and reflecting about the notions of cultural diversity, authorizes the family, with the group's help, to co-construct ways to think about the distress and the disease and to resolve the conflicts. These ways of thinking will in fact be *métissées* or hybridized, since migrant subjects, whether born abroad or of parents born abroad, are necessarily a *hybridization* of two cultures, that of their origins and that of their host country (50). The “understanding of cultural diversity” tends to predominate over “cultural competence” today (36).

The co-construction of meaning is a primary objective of the French transcultural pychotherapy approach. The cultural elements of the patients' attachments are not considered to be obstacles, but rather “active catalysts of the care relationship” (10). The patient's culture and various attachments are thus an integral part of the process of therapy. In this sense, the French transcultural approach is radically different from the models proposing to adapt the standard framework of care by training professionals in cultural competence (5, 13, 16, 17, 19, 22, 23, 31, 40, 51) or via integration through using interpreters or cultural mediators (35, 38, 52).

The objective is not to adapt the framework of care to the patients' particularities, but rather to change the paradigm of care. The treatment setting is organized to enable the emergence of cultural theories that explain the symptoms, and the recourse to traditional care by the patient and family, which will be the materials to work on in therapy. The introduction of cultural rationales into therapy and the acceptance and attribution of value to non-Western representations of distress and disease become important symbolic acts, because they belong to the families and to their history, and they can appropriate them or not, according to their desire and their pathway. The therapeutic relationship is thus rebalanced, more symmetric, because the family members put into this therapeutic relationship that which they consider important and which gives meaning to what is happening to them. It is no longer the therapist alone who decides what makes sense. The patient and family members have an active position in this therapy. The group of therapists and the family co-construct the meaning. Accordingly, real therapeutic work can occur; in a situation where it was previously impossible because of the asymmetry inherent in receiving a patient of a minority culture in a facility that symbolizes the majority culture of the host country.

Along the same lines, the models of training and liaison consulting (7, 8, 15–17, 19, 51), all involving advice to first-line therapists by transcultural experts, are limited by the adaptability of standard treatments. To the potential rigidity of the patient's psychological functioning is added the potential rigidity imposed by the type of supervision the patient's therapist receives. Transcultural psychotherapy, on the other hand, is addressed to the patient. Although the presence of the first-line therapist is encouraged to promote the patient's global therapeutic alliance, it is never compulsory, and the impossibility of working with the first-line team is never a *per se* limitation to the therapeutic work.

Finally, transcultural services are directed to all types of transcultural situations, unlike those aimed at specific symptoms, such as trauma (33, 34, 53) or at a specific type of population or ethnic group (27, 31, 35). The early data of a retrospective study underway show the clinical and cultural wealth of situations encountered (54).

## Strengths and Limitations

The scoping review approach presents some limitations. Nevertheless, the reason to choose this type of review was to permit the inclusion of a wide range of sources in order to present a broad overview of the available literature.

As a scoping review, information from a wide range of study designs and methods was gathered. The heterogeneity of the articles made it difficult to use an existing checklist to asses their quality, thus we ourselves created a simple tool to this end.

A broad search was necessary to collect all available information. To overcome this limitation, we used three different strategies for the literature search (systematic search for articles identified in scientific databases, direct questioning of our international network of transcultural therapists, and analysis of the abstracts of the principal journals in the field).

The final outcome of the review was not therefore an answer to a specific question, but rather a synthesized overview of the available literature.

## Data Availability Statement

The original contributions generated for this study are included in the article/supplementary material, further inquiries can be directed to the corresponding author/s.

## Author Contributions

All authors participated in every step of the conception of this paper, its writing, and its final approval.

## Conflict of Interest

The authors declare that the research was conducted in the absence of any commercial or financial relationships that could be construed as a potential conflict of interest.
